# Sensors for Detection of the Synthetic Dye Rhodamine in Environmental Monitoring Based on SERS

**DOI:** 10.3390/mi13111840

**Published:** 2022-10-27

**Authors:** Nguyen Tran Truc Phuong, Thuy-An Nguyen, Vu Thi Huong, Le Hong Tho, Do Thao Anh, Hanh Kieu Thi Ta, Tran Huu Huy, Kieu The Loan Trinh, Nhu Hoa Thi Tran

**Affiliations:** 1 Faculty of Materials Science and Technology, University of Science, Ho Chi Minh City 700000, Vietnam; 2 Vietnam National University, Ho Chi Minh City 700000, Vietnam; 3 Institute of Fundamental and Applied Sciences, Duy Tan University, Ho Chi Minh City 700000, Vietnam; 4 Faculty of Environmental and Chemical Engineering, Duy Tan University, Da Nang City 550000, Vietnam; 5 Department of Information Communication, Materials, and Chemistry Convergence Technology, Soongsil University, Seoul 06978, Korea; 6 Quy Nhon College of Engineering and Technology, Quy Nhon 590000, Vietnam; 7 Department of Industrial Environmental Engineering, College of Industrial Environmental Engineering, Gachon University, Seongnam 13120, Korea

**Keywords:** rhodamine, surface-enhanced Raman scattering (SERS), LSPR, sensor

## Abstract

This article presents a review of many types of SERS sensors for food safety and environmental pollution monitoring based on detecting rhodamine. It introduces the basic concepts of substrates, enhancement factors, and mechanisms, devices’ sensors integrated with the microstructure. Here, we review the state-of-the-art research in the field of rhodamine monitoring and highlight the applications of SERS sensors. The trends in the development of substrates for different applications have been mentioned with the aim of providing an overview of the development of different SERS substrates. Thus, an efficient approach for rhodamine detection has a good perspective for application in environmental monitoring.

## 1. Introduction

**Figure 1:**
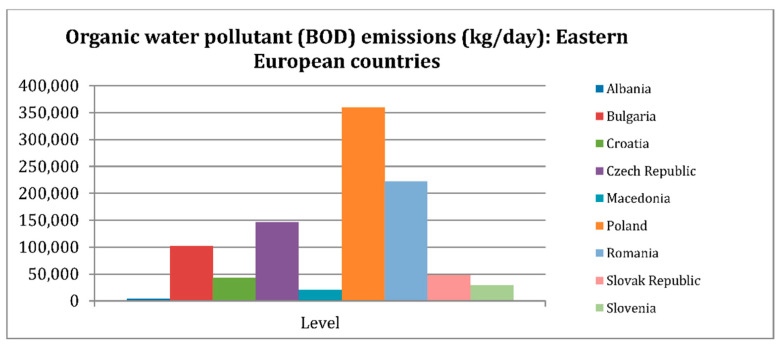
Water pollution caused by the textile industry of G20 countries, from a world database in 2007 (adapted with permission from [3], Copyright 2022, MDPI).

Every year, more than 7 × 10^5^ tons of synthetic dyes, containing more than 10,000 different dyes, are used worldwide [1]. It is estimated that in the dyeing industry alone, more than 200,000 tons of dyes have been leaked into the natural environment without being treated [2]. The textile industry is the leading cause of water pollution. Statistics by Paraschiv et al. [3] on the textile industry emissions of G20 economies (measured in percentages of total BOD emissions) (Figure 1) indicated concrete and convincing evidence that dyes from the textile industry are the main cause of serious water pollution. In Vietnam’s economy, the light industry, such as textiles, is now gradually asserting a leading role [4,5]. It helps bring great benefits and contributions to the economy. However, this also causes many negative impacts on the environment, especially the aquatic ecosystem, because a large number of dyes are released into the environment without treatment. There are many different color dyes used in the textile industry that have chemical compositions that often carry functional groups such as azo, anthraquinone, methine, nitro, arilmethane, carbonyl, and others. In addition, the high resistance of these pigments to light and heat makes their decomposition difficult [6]. This causes alarming harm to the environment and aquatic organisms. There are even some toxic chemical dyes that are capable of causing serious damage to the ecosystem even at levels as low as 1 mg/L [7]. Among them, dyes belonging to the rhodamine family, such as rhodamine B and rhodamine 6G, etc., are some of the dyes that cause many consequences not only for the environment but also for human health, even when present at extremely low concentrations.

**Figure 2:**
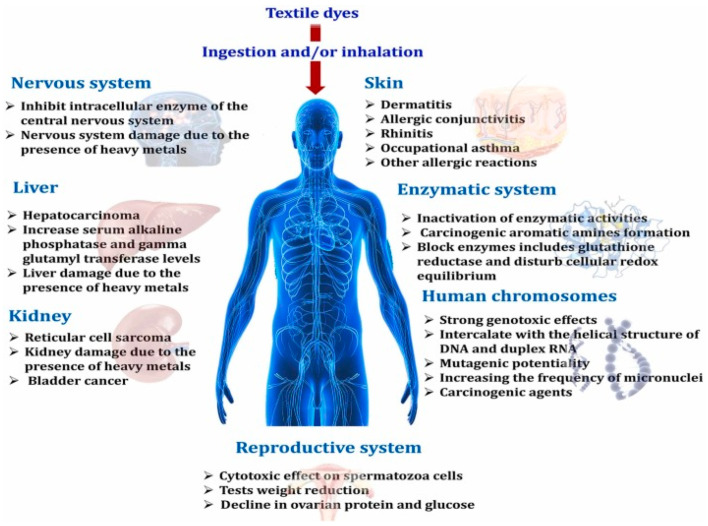
The negative effects of dyes on organs in the body. Adapted with permission from [9], Copyright 2022, Elsevier.

Besides the harmful effects on the environment, dyes belonging to the rhodamine family are also a serious threat to health when used in large amounts in coloring foods such as chili powder and melon seeds, etc. When accidentally ingesting rhodamine at high concentrations, there will be symptoms of poisoning, but the accumulation of rhodamine in the body also leads to more serious consequences, causing serious damage to the liver, kidneys, and even cancer [8]. As shown in Figure 2, there are a number of negative effects of dyes on various organs in the human body [9]. Therefore, Europe and most countries in the world have enacted an absolute ban on this substance in food.

Rhodamine is a dye that can be detected by a variety of methods, such as high-performance liquid chromatography (HPLC) [10,11], spectrophotometry [12], fluorimetric [13], enzyme-linked immunosorbent assays (ELISA) [14], electrochemical detections [15], surface-enhanced Raman scattering (SERS) methods [16,17], mid-infrared (MIR) spectroscopy [18], and near-infrared (NIR) spectroscopy [19]. HPLC is widely used and popular due to its high accuracy and ability to quantify chemical compounds, however, its complexity and flexibility make this method difficult to meet the requirements of diagnostics, clinical, rapid detection, and in situ performance [20]. ELISA is also a commonly used method for the detection of rhodamine, but the complexity of sample preparation and the large amount of analyte are also impediments to its application in assays and clinical diagnosis [21]. Overcoming those shortcomings, SERS has created a new step in sensors. The advent of handheld Raman sensor systems enables fast, sensitive detection at extremely low concentrations, on the molecular scale. The target substance can be detected from the original sample without going through the extraction or pre-denaturation process and toxic dyes are not used in this method.

SERS is an excellent solution to develop the outstanding properties of Raman spectral selectivity and completely solve the problem of signal magnitude and noise caused by significantly smaller Raman scattering cross-sections compared to fluorescence cross-sections. In 1974, for the first time, Fleischmann et al. used Raman spectroscopy to observe pyridine at different concentrations on a rough silver electrode background [22]. This has laid the foundation for the study of sensors based on Raman spectroscopy to detect organic substances at low concentrations. SERS can be seen as a result of this development. However, it was not until 1977 that SERS became known and received much recognition from the scientific community [23]. The leap in developing this method from the rough silver base to more complex morphological structures, such as metal nanostructure core-shells [24], alloys [25], multilayer structures [26], hollow [27], flower [28], etc., has been achieved through research and evidence. The dramatic improvement in the Raman signal makes SERS even more attractive, becoming a solution of the future with the ability to detect substances down to the molecular level. SERS basically makes the Raman signal intensity of an analyte molecule stronger even at very low concentrations by using materials with special effects. The enhancement factor (EF) is an extremely important parameter in evaluating the Raman signal enhancement ability of different materials. The EF can be used as a useful tool to study the physical properties of SERS, such as hotspots. However, the empirical methods for estimating EF are still controversial and not unified, although the concept of EF by SERS is clearly defined. In a study by Le Ru et al. [29], they pointed out the aspects related to EF in SERS. The article helps to solve problems in estimating EF experimentally and to clarify misconceptions about EF in SERS. The EF can be defined as the ratio of the SERS signal to the Raman signal that would be obtained for the same molecule in the absence of the SERS substrate under the same conditions. Comparatively, the EF can be calculated based on the ratio of the measured Raman intensity of the analyte on the SERS substrate at a known concentration and the normal Raman intensity of that analyte. However, it should be ensured that all conditions during measurement, such as laser, microscope, and other parameters, are kept constant. Such normalization is called the analytical EF (AEF) and is determined by the formula:

$$\mathrm{AEF}=\frac{I_{\mathrm{SERS}/C_{\mathrm{SERS}}}}{I_{\mathrm{Rs}}/C_{\mathrm{Rs}}}$$

However, this calculation still has many flaws because the absorption on the substrate surface of different analytes will lead to differences. At the same time, methods of slicing or spinning the analyte onto the matrix do not guarantee the same density. Therefore, another way to normalize EF is called SERS substrate EF (SSEF), which is determined by the formula:

$$\mathrm{AEF}=\frac{I_{\mathrm{SERS}/N_{\mathrm{Surf}}}}{I_{\mathrm{Rs}}/N_{\mathrm{Vol}}}$$

where N_Vol_ = c_RS_V_Sca_ is the average number of molecules in the scattering volume (V_Sca_) for the Raman (non-SERS) measurement. However, the challenges involved in determining the number of molecules on the surface have become a barrier in this method.

Many theories have been put forward to explain the SERS phenomenon, but to this day, there are still many controversies surrounding this issue. The most accepted theory is the electromagnetic enhancement mechanism. This hypothesis assumes that the Raman signal enhancement comes from local fields generated on the surface of precious metal nanoparticles by the localized surface plasmon resonance (LSPR) effect, that has been proven to be the main enhancing mechanism in SERS with up to 10^6^–10^8^-fold enhancement potential [30]. Plasmon is a concept that refers to the collective vibrations of free electrons that exist in precious metals that are explained by classical physics [31]. Alternatively, the plasmon is the collective oscillation of the free electrons with respect to the fixed positive ions in the metal. Localized surface plasmons result from the confinement of the surface plasmon in a nanoparticle equal in size to or smaller than the wavelength of light used for plasmon excitation [32]. Usually, the oscillations are quickly suppressed by defects or by the lattice nodes themselves in metal. However, when the size of the metal is smaller than the incident wavelength, the quenching phenomenon is no longer available, but the electronics will oscillate and respond to the stimulating light. This explains that the precious metals in the bulk form will not observe this phenomenon, or the intensity is very small depending on the bulk size. For thin-film materials of precious metals, the electron density on the surface is very large. When stimulated by incident light, these electron clouds will vibrate and propagate along the membrane surface, generating a strong electromagnetic field on the surface near the membrane, which is known as the surface plasmon resonance (SPR) effect. The electric field at the analyte surface increases the Raman polarization, which leads to an increase in the Raman emission power. This electric field is mainly generated by the LSPR or SPR effect generated by the SERS substrate [29]. Therefore, precious metal nanomaterials characterized by strong LSPR effects have attracted much interest in SERS. The signal is further enhanced when it occurs in the gaps, crevices, or sharp features of plasmonic materials because it is here that the electromagnetic field is strongest. For well-optimized surfaces, the EF is also possible up to 10^10^–10^11^ times [33]. Another mechanism is also the enhanced Raman signal in SERS, which revolves around the electron transfer between the SERS-enhanced substrate and the analyte through chemical bonds. The charge-transfer electrons in different states resonate with the incident wavelength. Enhancing the Raman signal of the analyte molecule oscillations is referred to as the chemical (CM) mechanism [34,35]. These mechanisms are very wavelength-dependent on the Raman excitation and are difficult to identify and demonstrate for their contribution to the enhancement of the Raman signal. On the other hand, the electromagnetic field generated from the plasmon is so large that electromagnetic (EM) becomes the main enhancing factor in the mechanism of SERS. The enhancement coefficient from the local fields of the metal nanoparticles is proportional to the factor equal to the fourth power of the electromagnetic field, which is the main reason for the substantial enhancement of EM [36,37,38]. So far, SERS has been studied for many different applications, especially in sensors to detect biological molecules, proteins, DNA, organic substances, detect cancer, etc. Development of the power of SERS opens up more intensive development directions from precious metal nano-subsets such as Au and Ag with plasmonic regions in the visible and near-infrared regions to the development of UVSERs using plasmons of transition metals such as Pd/Pt, Ru, Rh, Co, and Al. The obtained results show promise for the application of SERS in the detection of biomolecules with electronic resonance in this wavelength region. However, the development of UV-SERS has also encountered difficulties due to the potential of UV rays to cause optical degradation of the analyte or matrix. In addition, the strengthening ability of metals is also recorded at a low level of about 250 times. For that reason, the choice of the appropriate substrate and excitation wavelength is an extremely important factor in the success of this method.

SERS is a potential method with high applicability in many research fields. At present, there are still many mysteries surrounding the mechanism and extent of enhancement of this method, so having an overview is necessary for further research and development directions for improvements for SERS. This paper briefly summarizes the progress developed in improving the sensitivity of SERS for the detection of rhodamine B and other members of the rhodamine family. This is a common organic substance used in industry, which causes many consequences for the environment and human health.

## 2. The Process of Developing the Material Structure of the Substrate by the Electromagnetic (EM) Enhancement Mechanism

### 2.1. SERS Is Based on the Homogeneous Material

The trend of applying precious metal nanoparticles with different shape structures has been promoted, and a series of research works using single-component metal nanoparticles for SERS applications have been carried out and obtained many desirable results due to the stability, simplicity, and controllability of the material. In this section, homogeneous metals express surface plasmon resonance (SPR), and LSPR effects that enhance electric fields such as Au [16,39], Ag [40,41], etc., are widely studied, which are effective substrates for SERS detection. The roughened nano-Au film coatings have been made simple and fast by electrochemical methods for the rapid detection of R6G molecules. The coating thickness parameters were investigated corresponding to detection limits at approximately 10^−11^ M [16]. The solution of colloidal gold particles is applied in the SERS sensor with different approaches, from ordinary colloidal gold particles to polystyrene-coated gold colloidal particles and gold colloidal particles annealed from 200 to 400 °C. This experiment shows potential promise in the application of colloidal gold particles in the selective detection of R6G and crystal violet at high temperatures [39]. Citrate-coated Ag nanoparticles were used as substrates for direct rhodamine B detection assays on chili powder using a handheld Raman spectrometer. This study is a step forward in putting SERS into practice. The silver nanoparticle substrate exhibits an enhancement factor of up to 1.6 × 10^7^ times at a wavenumber of 625 cm^−1^. The calculated detection limit corresponding to a linear operating range of 0.2–20 µg/L is 0.08 µg/L [40].

One of the most prominent representatives of the metals of interest, with a focus on applied research, is silver. Strong LSPR strength, good binding ability to surfaces through thiolate and amine functional groups, and good antibacterial properties are the factors that make Ag a bright star in the selection of metals for SERS applications to detect rhodamine B. Metal nanoparticles fabricated from basic morphologies such as the sphere [40,42], plates, nano-decahedra [43], wires [44,45], etc., to more complex morphologies such as cube [45,46], pyramid [47], star [48], flower [49,50], dendrites [51], etc., were investigated for rhodamine.

**Figure 3:**
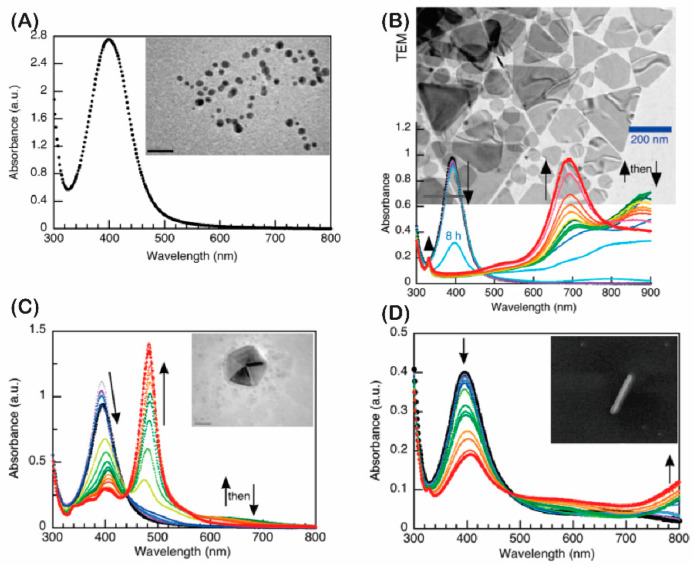
The change absorbance conversion during irradiation (initial in black, final in red) of UV−vis spectrum with irradiation time and TEM image shows the morphology of the particles formed. (**A**) The AgNP seeds after UVA irradiation, (**B**) 627 nm LED excitation of AgNP seeds, (**C**) 455 nm LED excitation of AgNP seeds, and (**D**) 720 nm LED irradiation. Adapted with permission from [57], Copyright 2010, American Chemical Society.

Many different methods have been used to fabricate Ag and Au nanoparticles, such as electrochemistry [52], intermediate nucleation [53], hydrothermal [54], and irradiation [55,56]. The chemical reduction method and irradiation method are chosen for many applications in SERS for detecting dyes because of the simplicity of the equipment, and the quick and convenient process of synthesizing and manufacturing materials. By varying the time or wavelength of irradiation, silver nanoparticles with different morphologies were formed, which is one of the reasons this method is so appreciated. In this way, different morphologies were produced, as reported by Stamplecoskie et al. [57]. The plasmon peak shift corresponds to the morphological change made by varying the time and wavelength irradiation, as shown in Figure 3. The flexible application of reducing agents and different reduction mechanisms creates a variety of morphologies of the nanoparticles, which improves the SERS enhancement of the substrate. Commonly used reduction methods are reduction with NaBH_4_, citrate, mirror reaction, and the shape control polyol method, etc. Some studies on the fabrication of silver nanoparticles by the chemical reduction method can be mentioned, as reported by Liang et al., which showed a good ability to enhance the rhodamine B signal in chili powder. In this experiment, the Ag NPs solution has been shown to have a high sensitivity, with a detection limit of 0.08 µg/L which is calculated in an investigation area of 0.2 to 20 µg/L, with a standard deviation of no more than 4.84% [40]. Many studies show that allowing metal nanoparticles to disperse in solution does not guarantee high signal stability and the enhancement ability is not promoted in the best way. That is why more and more research is focused on immobilizing metal nanoparticles on substrate surfaces to ensure the stability of SERS-enhanced substrates. In a previous study of ours [46], investigating the rhodamine B detection ability of silver nano-cubes showed a 100-fold better enhancement when the nanoparticles were immobilized on the glass substrate through the functional group −NH_2_ compared with when AgNCs were dispersed in DI. The reason is that the control over the distribution and density of particles is better, so the stability and enhancement coefficient is improved. The limit of detection (LOD) of AgNCs substrates immobilized on glass up to 10^−10^ M was calculated from a linear range of 10^−5^ to 10^−10^ M corresponding to the correlation coefficient R^2^ = 0.99. The levels of morphological complexity used for the purpose of increasing the enhancement factor in SERS are accomplished with many achievements. The tendency to create pointed structures has been selected and obtained many results. The report by Barveen et al. [49] on the enhancement ability for rhodamine 6G (R6G) and Congo Red (CR), both separately or in a mixture, showed good enhancement of the Raman signal. In this report, the author used a combination of silver nanoparticles with silver nanoflowers to create a perfect electromagnetically enhanced structure with dense hotspots and nanogaps on the nanoflowers and between the nanoparticles to form an ability to enhance and capture analytes. The detection limit of R6G on substances that should be combined with AgNPs and AgNFs reaches 10^−14^ M when tested alone and 10^−13^ M when tested simultaneously for R6G and CR, with homogeneity < 10.27%, respectively, corresponding to an enhancement factor of up to 10^12^ times.

### 2.2. SERS Is Based on the Heterogeneous Material

**Figure 4:**
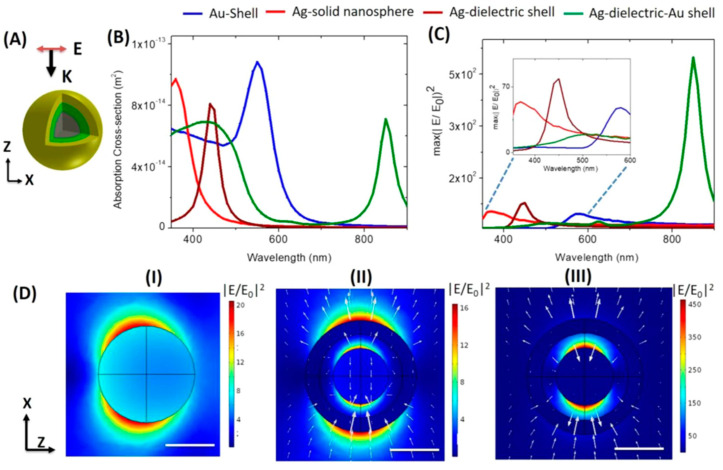
(**A**) HMCS structure consisting of an inner gray solid silver metal followed by a green silica dielectric layer and an outer gold shell. (**B**) The absorption cross–section of HMCS structure comparison. (**C**) The different cases of enhancements (maximum |E/E_0_|^2^ in the entire volume). (**D**) Electric field distribution diagrams of solid metal nanoparticles (**I**), core–shell metal nanostructures (**II**), and HMCS (**III**) structures, respectively. Adapted with permission from [59], Copyright 2019, Springer Nature.

The distribution of the electric field on the surface of metal nanoparticles depends on the resonance frequency of the hybrid metal nanoparticles. Electromagnetic intensity is highest at the interface because the dipole generated from the core causes the charge to concentrate on the outer part of the core. Similarly, the electric dipole caused by the shell material also concentrates some of the charges on the inner surface of the shell. The dipole–dipole coupling causes the contact surface between the two materials to reach the maximum enhancement of the electric field, that is caused by the accumulation of positive and negative charges at the antisymmetric mode of dipole plasmon resonance. On the contrary, for the symmetric mode at the resonant frequency, the highest electric field enhancement concentrated on the surface of the core and the top of the Ag shell. Charges of the same sign are concentrated on both the inner and outer surfaces of the shell, thereby causing this maximum electromagnetic field enhancement on the surface of Au/Ag particles, as reported by Zhou et al. [58]. The silver core−dielectric shell−gold shell (HMCS) structure has been compared with the solid metal single structure, and the core–shell structure reported by Paria is presented in Figure 4 [59]. Changes in the absorption cross-sections of different structures can be observed from the solid silver sphere, Au-shell, Ag-dielectric, and Ag−dielectric−Au shell (Figure 4B). This result proves that there is an interaction between the metal core layer and the shell, leading to the peak separation. The electric field vector plot and spatial distribution of the local spatial enhancement of the solid silver nanocluster (Figure 4D(I)) and the HMCS structure in the antibonding mode (Figure 4D(II)) and the binding mode (Figure 4D(III)) indicates that the HMCS structure has an enhancement arising both at the outer surface of the gold shell and the inner dielectric pad, and the increase in strength is strongly limited to the silica shell.

Precious metal nanoparticles are fabricated in combination with each other or with other metals to heterogeneous materials with different morphologies that have been demonstrated for good enhancement in SERS. In addition, the purpose of combining Au and Ag with other metals also meets the needs of the cost of materials, increased morphological stability, or protecting precious metals from external agents to help increase the strength of the base material. Based on that, many structures that combine metals together have been carried out for the purpose of detecting rhodamines, such as the Au@Ag core−shell structure made by Wu et al. [60]. It has been demonstrated that the electromagnetic field coupling of Au and Ag metals affords an excellent enhancement effect to SERS. A variety of organic compounds were used to investigate, and rhodamine (R6G) showed a good signal when tested at 10^−13^ M [60]. Following previous studies, the Au–Ag double-shell structure was developed by Wei et al. to detect rhodamine B in chili powder. The direct detection of chili powder through simple sample processing of the double−shell Au–Ag substrate successfully detected 5 µg/g of rhodamine B, demonstrating the stable detection ability of suitable SERS substrates for clinical rapid detection applications [61]. In addition to combining the electromagnetic fields of Au and Ag metals, Au with a stable structure is often used as a framework to synthesize AgNPs with special morphology, with spikes that help enhance the SERS of the substrate. The star−shaped nanoparticles with the Au and Ag layers are highly concentrated on the end of the tips of stars designed by Child et al. This structure helps to focus the strong electromagnetic field on the spikes, where the SERS signal will be greatly enhanced. A test for R6G that investigated the enhancement capacity of this structure showed an enhancement factor of up to 10^5^−fold with the presence of most of the characteristic C–C–C ring in the in−plane oscillations vibration mode (C–H) at 610 cm^−1^, out−of−plane bending mode at 774 cm^−1^, ν(C–H) in−plane bending mode at 1130 and 1578 cm^−1^, N–H in−plane bending mode at 1313 cm^−1^, and ν(C–C) stretching mode at 1364 and 1509 cm^−1^ [62]. In addition, metal−organic frameworks (MOFs) are also an interesting option because the surface area is significantly increased, and they act as cages for the efficient capture of analyte molecules. When combined with nanoparticles such as Au or Ag, it will help make the most of the electromagnetic field generated by these particles. Ag nanoparticles combined with CuFe_2_O_4_, which are morphologically simple MOFs affording excellent efficiency to the 6G rhodamine analyte, are presented in a report by Kamal et al. [63]. In this report, the author has succeeded in 6G rhodamine detection with a detection limit of 10^−14^ M corresponding to EF up to 10^12^ times due to the synergistic effect resulting from uniform Ag distribution on the cubic morphology, leading to the high electromagnetic effect and chemical mechanism of CuFe_2_O_4_ [63].

## 3. Developing the Material Structure of the Substrate by the Chemical Mechanism (CM)

**Figure 5:**
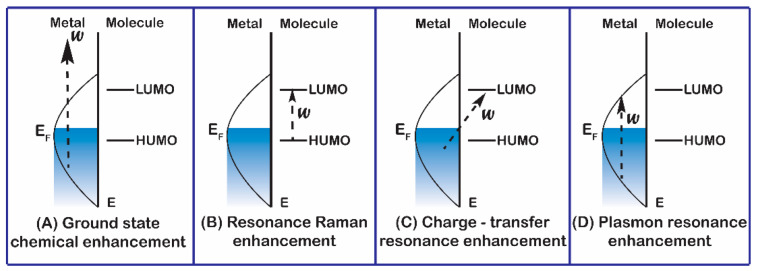
Schematic depiction of chemical enhancement mechanisms applied in SERS [35].

**Figure 6:**
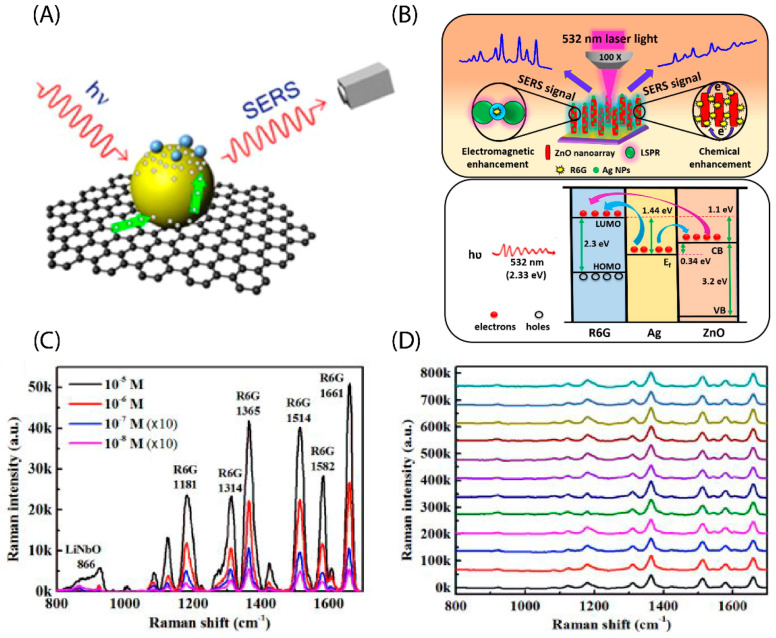
Schematic depiction of the enhancement mechanism of the substrate (**A**) graphene/Ag. Adapted with permission from [64], Copyright 2013, Elsevier. (**B**) ZnO/Ag, (**C**) Raman spectrum of R6G on ZnO/Ag heterogeneous substrate from 10^−5^ to 10^−9^ M concentrations, and (**D**) Raman spectrum of 20 random locations at a 10^−5^ M R6G concentration on ZnO/Ag substrate. Adapted with permission from [65], Copyright 2020, Elsevier.

Besides the electromagnetic enhancement mechanism, another mechanism, the chemical mechanism, also contributes greatly to the enhancement of the SERS signal. Although the strongest signal enhancement still comes from the electromagnetic mechanism, the combination of these two mechanisms shows significant improvements not only for the enhancement factor but also for other factors such as sensitivity and stability. The chemical enhancement mechanism can include enhancement cases as depicted in Figure 5. First, the enhancement can take place at the ground state due to the interactions of the nanoparticles and substance molecules analyzed, and this was not related to any external stimuli to the system (Figure 5A). Next, the enhancement associated with the excitation wavelength matches the transition of the molecule from the HUMO level to the LUMO level, namely the resonance of the excitation wavelength with the molecular transition (Figure 5B). Finally, charge transfer occurs when the excitation light has energy suitable for the electron transfer from the nanoparticles to the analyte molecule. In other words, resonance occurs between the excitation wavelength and the nanoparticle–molecule charge−transfer transitions (Figure 5C). Semiconductors and graphene derivatives are the ones that have received a lot of attention, as their combination with metal nanoparticles shows results that exceed expectations. Both graphene and semiconductors are capable of enhancing Raman signals through various mechanisms, such as surface plasmon, tunable bandgap, charge transfer, exciton, and molecular resonance. In particular, the easy adjustment of the conductivity, bandgap, and even the refractive index of the semiconductor is also a factor that can be exploited and tailored to the analyte to obtain the best chemical enhancement results. Kong and colleagues fabricated the SERS−enhanced substrate by combining graphene with gold nanoparticles, and the results obtained from this trial demonstrated graphene’s assistance in enhancing SERS by approximately 2−fold compared to that of individual Au nanoparticles. SERS substrate structure diagram is depicted in Figure 6A. The enhancement mechanism is also proposed whereby the free electrons on the surface of graphene are transferred to the 6 s orbital of Au atoms, which causes the Au 6 s energy level to be raised, increasing the possibility of electrons from the Au surface to the LUMO of the R6G molecule. In addition, the accumulation of the rich delocalized π electrons on the graphene surface also causes the aggregation of the upper surface electrons of the gold nanoparticles, whereby both EM and CM mechanisms are enhanced [64]. The heterogeneous ZnO/Ag structure has been successfully synthesized by a green method using ferroelectric templates made by Wang et al. [65] (Figure 6B–D). They demonstrated the possibility of development in the use of combinations of materials to create the heterogeneous nanostructure to take full advantage of the SERS enhancement effect. Based on electron transfer from the Fermi level of Ag to the HUMO of R6G or from the conduction band of ZnO to the HUMO of R6G, the combination with Ag makes the electron transfer from ZnO to R6G easier because the conduction band of ZnO is electronically enriched by an excitation wavelength of 532 nm, which has helped the electrons of Ag be transferred to the conduction band of ZnO [65].

## 4. Influence of Substrate Morphology and Material on SERS Application

**Figure 7:**
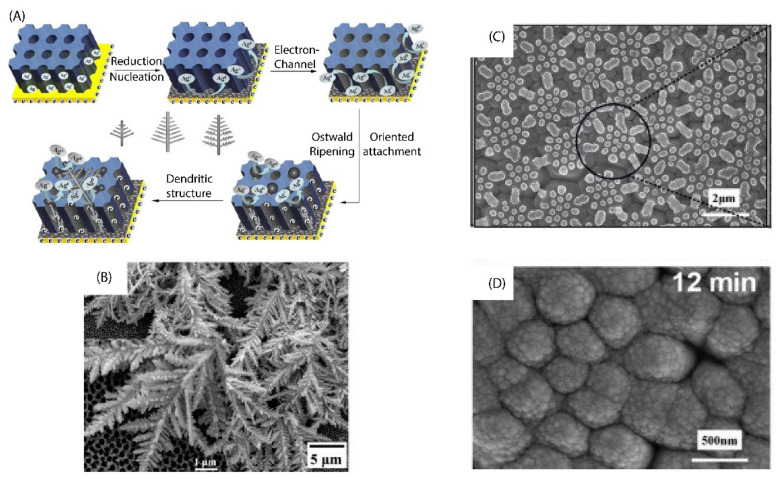
(**A**) Diagram depicting the development of silver dendritic on the AAO substrates, and (**B**) FESEM image of silver dendritic on AAO substrates. Adapted with permission from [69], Copyright 2016, Elsevier. (C) FESEM image of PDMS surface after copying from the diatom, and (**D**) FESEM image of PDMS surface reproduced from diatoms after creating a sputtering Au coating. Adapted with permission from [70], Copyright 2020, Elsevier.

The distribution of substrates is also an important factor that greatly influences the extent of SERS enhancement of the substrates. The solution of precious metal nanoparticles, after being successfully synthesized, can be used directly to detect rhodamine; however, many studies have shown that this method is not the most effective when it is challenging to control the Raman signal of the analyte molecule, which is caused by Brownian motion of particles in solution. Therefore, many methods have been proposed to immobilize these metal particles to improve the stability and increase the enhancement coefficient of the SERS substrate. Self−assembled monolayer coating assays of nanoparticles are proposed as a solution to the stability problems and to increase detectability for rhB [46]. In addition, based on theories of electromagnetic enhancement, many different morphological substrates have been investigated. Silicon wafers are very popularly used to make SERS substrates because they easily modify the surface to attach the NPs or create different structures on the surface. The pyramid structure is the most used structure as it optimizes the hotspot effect, which significantly increases the SERS efficiency thanks to the narrowed nanoparticle spacing of less than 20 nm. This uneven structure also notably increases the contact surface and the number of warming spots, that is for the SERS properties of the substrate to be significantly increased, as reported by Wang et al. [66]. In addition, there are still more complex structures made by techniques such as AAO, photolithography, nanoimprint lithography, etc. The structures built from these techniques are distinguished by high uniformity, and the nanometer size creates extremely dense hotspots (Figure 7). One of the examples of the enhanced capacity of these nanostructures was a report by Lee et al. [67], where the detection limit for R6G reached 1 fM, corresponding to an RSD < 8%. In this study, the structure of nanogaps is about 3.4 nm between Ag arrays. This is the result that is close to the best distance condition from the calculated model [68].

Another direction of research in the development of SERS substrates is the use of periodic plasmonic structures. The plasmonic material is designed with sequentially arranged structures to support Raman signal enhancement in the proposed rhodamine detection application. A good example is the sputtering fabricated gold nanomembrane structure, which is designed in the shape of sequential pyramids under the shaping support of the Si substrate. In this study, the Si substrate was created with a pyramidal structure by an anisotropic wet−etching method. From the 3D-FDTD simulation, it is shown that this structure generates concentrated hotspots on the pyramid’s vertices. The EF of this SERS substrate structure for R6G reached 3.8 × 10^5^ times, calculated at the thickness of the gold film of 125 nm reported by Rui Li et al. [71]. Multiscale three-dimensional (3D) structures copied from CD−R tracks have been reported by Zhou et al. [72]. In this study, the authors created ZnO nanorods on top of the groove structure copied from the surface of CD−R using polydimethylsiloxane (PDMS). Then, a layer of Ag nanoparticles was coated on this structure by a thermal evaporation method. In the enhancement of the Raman signal generated in addition to the enhancement by the EM mechanism of Ag and CM mechanism of ZnO nanorods, the 3D groove structure is thought to contribute a significant part. The 3D structure helps to confine the excitation light by multiple scattering between the tracks. At the same time, it increases the contact area of the substrate. The result obtained based on this construct is a LOD of 1 × 10^−11^ M and a high EF of 7.0 × 10^8^ for rhodamine 6G (R6G) as the probe molecule.

Some argue that rigid bases are not very flexible in their application in real-life samples for clinical detection applications that directly sample the facets. Therefore, many proposals on switching from using rigid substrates such as Si, SiO_2_, etc., to flexible substrates from materials such as PET, PDMS, and tape, etc., are widely applied as these materials can be bent, allowing easy integration on machine probes and better contact with rough surface samples. Several studies have also shown that the polymer structure helps to better absorb the analytes, which in turn improves the SERS properties. The SERS microfluidic chip has been designed by Gutiérrez by coating a silver layer over a layer of cyclic olefin copolymers and an ultrathin nanostructured polyethylene terephthalate foil. In addition, a sufficiently thin PET layer was coated on the surface of Ag to limit the fluorescence signal, which is responsible for the Raman signal degradation, successfully demonstrating the sensor’s Raman enhancement factor of 10^5^–10^6^-fold [73]. Polydimethylsiloxane (PDMS) is known to have high strength, high transmittance, a low Raman cross–section, and good flexural strength, so it is widely used in the fabrication of flexible SERS substrates. Inspired by the flexible bending ability of PDMS, Mao and colleagues fabricated nanoscale metallic cracks on the PDMS surface. Accordingly, with certain bending angles, the metal patterns on the surface help the enhanced Raman signal reach 4 × 10^6^-fold for rhB, which shows high compatibility and application flexibility for different probes [74]. The research works representing the development directions of SERS substrates in detecting dyes belonging to the rhodamine family are listed in Table 1.

## 5. SERS Approaches to Rhodamine Detection for Food Applications

There are currently no commercially available integrated devices or products using SERS for the quantification of analytes. In addition to studying SERS materials and investigating the ability to enhance the signal when the analyte is isolated, experimental studies on real samples have been investigated and demonstrated the ability to detect dyes in the environment field with interference. Although rhodamine is a potentially cancerous poison and is banned from being used in food at all concentrations, thanks to the advantage of its low cost and beautiful color, chili powder production facilities are still allowed to use it as a food coloring. There are many methods to detect the existence of rhodamine B, such as HPLC. However, for the needs of food testing, clinical evaluation requires a simpler, less expensive method and a fast response time. Therefore, the SERS test is an optimal choice for this application. Many studies have tested directly on commercial chili powder samples with positive results with high accuracy, such as in a report by Lixia Zhang et al. [44], who proposed a procedure using surface-enhanced resonance Raman scattering (SERRS) on silver nanowire substrates. Chili powder, chili sauce, and Chinese prickly ash purchased from the market were extracted by ethanol and then dispersed into DI before being processed for analysis. The purpose of this dispersion is to help the analytes to be evenly distributed and better absorbed on the surface of the substrate compared to keeping them as a powder or sauce. In this study, relevant influencing factors such as the pH of the analyte solution, contact time of the rhB solution, and the substrate. were investigated. The limits of detection (LODs) for rhB were 0.35 µg/g in chili powder, 0.14 µg/g in chili sauce, and 0.02 µg/g in Chinese prickly ash. The relative standard deviations (RSD) were between 2.18% and 4.56% and accuracy was in the range of 80.0–98.7% for different spiked food products. Similarly, another publication by Sun et al. [79] also reported on testing rhodamine B in chili powder samples. In order to ensure a stable signal, these chili powders must undergo pretreatment steps according to the procedure reported by Li et al. [80] before being applied to the SERS substrate. The study demonstrated the ability to detect rhodamine B in chili powder at a concentration of 5 µg/mL on an Au nanorod-incorporated melamine foam substrate. The reliability of the results was checked by the authors via HPLC analysis. From the above positive results, the application of SERS in the detection of rhodamine in food products is completely possible. However, the challenge for the development of SERS sensors in food is the ability to quantify the concentration of analytes and the influence of interferences and to standardize the extraction procedure.

## 6. Conclusions

This paper provided an overview of the application of surface-enhanced Raman scattering for the purpose of detecting rhodamine dyes. The selection of materials, or the structure of the substrate, is thus easier to be selected for different purposes of application, such as in food, the environment, etc. In addition, understanding and covering the enhancement mechanism and material properties, structure, morphology, and factors affecting the detection ability of rhodamine dye plays an important role in obtaining results and directing the development of research. The next step is to quickly put the results achieved into practical applications. At the same time, this is also a knowledge base to overcome the limitations of devices applying Raman technology in current sensors. Unauthorized or leaked rhodamine dyes will be detected more quickly and promptly when these substrates are optimized and successfully integrated into simple handheld Raman devices.

## Figures and Tables

**Table 1:** A comparison of SERS substrates has been used to detect rhodamine dye.

Substrate	Synthesis/Fabrication	Analytes	Linear Range	EF (Fold)	LOD	Ref
Nano-Au film	Electrochemical	R6G	10^−5^–10^−9^ M	10^8^	10^−11^ M	[16]
Ag NPs solutions	Chemical reduction method	RhB	0.2–20 µg/L	1.6 × 10^7^	0.08 µg/L	[40]
AgNFs @ AgNPs	The one-step solution-phase	R6G	-	10^12^	10^−14^ M	[50]
ITO/Ag NDs	Electrochemical	R6G	10^−5^–10^−11^ M	1.3 × 10^6^	10^−11^ M	[69]
Au@Ag NCs	Liquid–liquid self-assembly and evaporation-induced self-assembly methods	R6G	10^−8^–10^−12^ M	2.2 × 10^8^	10^−12^ M	[75]
ZnO/Ag heterogeneous	Hydrothermal, photoreduction	R6G	10^−5^–10^−8^ M	7 × 10^8^	<10^−8^ M	[65]
Graphene/Ag NPs on multilayer film	Vacuum thermal evaporation, CVD	R6G	10^−6^–10^−11^ M	2.8 × 10^7^	10 ^−11^ M	[76]
Flexible AgNPs/AgNPs-PDMS	Chemical reduction method, dip-coating method	R6G	10^−9^–10^−13^ M	8.3 × 10^9^	10^−13^ M	[77]
CuFe_2_O_4_ (Ag-CFO) microtubes	MOF template	R6G	10^−5^–10^−13^ M	10^12^	10^−14^ M	
Ag@CoFe_2_O_4_/Fe_2_O_3_ nanorod arrays	Hydrothermal and photochemical reduction process	R6G	10^−8^–10^−10^ M	1.2 × 10^8^	10^−10^ M	[78]

## Data Availability

Not applicable.
